# How to Effectively Identify Patients With Rifampin-Resistant Tuberculosis in China: Perspectives of Stakeholders Among Service Providers

**DOI:** 10.3389/fpubh.2021.736632

**Published:** 2021-11-24

**Authors:** Qianhui Hua, Hong Xu, Xinyi Chen, Junhang Pan, Ying Peng, Wei Wang, Bin Chen, Jianmin Jiang

**Affiliations:** ^1^School of Medicine, Ningbo University, Ningbo, China; ^2^Department of Tuberculosis Control and Prevention, Xiaoshan District Center for Disease Control and Prevention, Hangzhou, China; ^3^Department of Tuberculosis Control and Prevention, Zhejiang Provincial Center for Disease Control and Prevention, Hangzhou, China; ^4^Key Laboratory of Vaccine, Prevention and Control of Infectious Disease of Zhejiang Province, Hangzhou, China

**Keywords:** rifampin-resistant tuberculosis, screening, policy, stakeholders, attitudes

## Abstract

To evaluate China's current rifampin-resistant tuberculosis (RR-TB) screening strategy from stakeholders' perspectives, the perceptions, attitudes, and interests of 245 stakeholders from three eastern, central, and western China provinces on RR-TB screening strategies, were investigated through stakeholder survey and interview. The attitudes toward three RR-TB screening strategies were statistically different: inclination to choose who to screen (*Z* = 98.477; *P* < 0.001), funding for rapid diagnostic technology screening either by reimbursed health insurance or directly subsidized financial assistance (*Z* = 4.142, *P* < 0.001), and respondents' attitude during RR-TB screening implementation levels (*Z* = 2.380, *P* = 0.017). In conclusion, RR-TB screening scope could be expanded by applying rapid diagnostic technologies. Provinces with different economic status could adjust their screening policies accordingly.

## Introduction

Tuberculosis (TB) is an infectious disease that seriously endangers public health and one of the major infectious diseases in China. It is a major disease that causes poverty and restricts economic and social development globally, including China (1). According to the 2020 World Health Organization (WHO) Global TB Report, there were ~833,000 new TB cases in China in 2019, with an incidence of 58/100,000, which indicates that the burden of TB in China remains high (2, 3). TB adversely affects patients' psychological, economic, and social well-being and therefore reduces the quality of life (4). TB patients generally have poor economic conditions, and people affected by TB often face economic hardship, vulnerability, marginalization, stigma, and discrimination (4–7).

In recent years, the emergence and prevalence of rifampin-resistant TB (RR-TB), which is a great challenge to the prevention and control of TB (8). China has made substantial progress in TB control over the past two decades and has more than halved the TB prevalence (9). Currently, TB control depends largely on two aspects: individual patients' behavior of reporting the symptoms to the health care department and the effectiveness of the health system in correctly identifying and diagnosing patients once they reach their provider network (10). There are two main methods for RR-TB screening: antimicrobial susceptibility testing (AST) and rapid molecular detection; the molecular detection method plays a pivotal role in the identification of drug resistance in pathogenic bacteria. From 2017 to 2019, with the gradual increase in the coverage of RR-TB screening, the number of people receiving RR-TB treatment reported in China increased by more than 10% (2). However, because of China's vast territory and significant differences in culture, economy, geography, and infrastructure, the prevalence of TB varies from region to region. One study (9) showed that the gap between eastern, central, and western regions has widened; both the absolute value and the percentage change in the prevalence of bacteriologically positive TB in the central and eastern regions were greater than those in the western regions. Western China is the region with the highest TB prevalence rate, and it is lagging in reducing the burden of the disease. Different regions have varying levels of financial resources, numbers of well-trained health care workers, public health infrastructure capacities in terms of solving TB problems and so on, which will affect the policy of the RR-TB screening.

At present, the screening of RR-TB in China is still at its early stage, with no fixed pattern or standardized mechanism in place. Due to a variety of factors including the lack of RR-TB screening capabilities (especially for the rapid molecular detection) in many county-level designated hospitals, the lack of inclusion of diagnostic costs for reimbursement, and the long diagnostic interval, a large proportion of RR-TB patients experience delayed diagnosis and cannot be tested for drug resistance in a timely manner, possibly resulting in more opportunities for community transmission.

The screening strategies for RR-TB varies greatly from province to province in China. There are few empirical studies on health providers' perspectives on the challenges that they face in RR-TB detection at implementation level in China (11). Future progress in RR-TB may depend on the ability of providers to appropriately detect and refer TB patients for further care (12). Furthermore, stakeholders among service providers have different opinions about the methods and models of screening. Some studies have shown that efforts to expand services tailored to transhumance patterns and diagnostic capacity at primary healthcare unit levels, should be prioritized (13). Therefore, an appropriate screening strategy for rifampin-resistance should be explored.

This study aimed to evaluate the current screening strategies for RR-TB in China from the perspectives of stakeholders among service provider, and to provide recommendations based on scientific evidence, for the sustainable development of the screening strategies for RR-TB.

## Materials and Methods

### Definition of the Stakeholders

From October 2018 to March 2019, we collected and reviewed literature and policy documents about MDR-TB prevention and control from the Chinese internet literature database, and based on the stakeholder analysis guidelines, an initial candidate stakeholder list was developed (14). Ten experts working in the national and provincial institutes of MDR-TB programs were invited to judge whether these candidate stakeholders should be considered as stakeholders. Finally, 19 kinds of stakeholders were recognized. In this survey, 15 types of stakeholders among the service providers were identified for inclusion. After a panel discussion, the stakeholders were integrated into three major categories (Table 1): (1) service policy implementers (medical and nursing department of prefecture-level TB designated hospital, management department of prefecture-level TB designated hospital, management department of county-level TB designated hospital, medical and nursing department of county-level TB designated hospital, prefecture-level laboratory testing department, community follow-up management department, county-level laboratory testing department, department of TB prevention and control of provincial center for disease control and prevention (CDC), department of TB prevention and control of county-level CDC, department of TB prevention and control of prefecture-level CDC), (2) policy makers (administrative department of health, department of medical insurance, administrative department of civil affairs, administrative department of finance), (3) others (charitable non-governmental organizations (NGOs) such as the Red Cross, manufacturers). The staff from the corresponding department were considered as the study subjects in this study.

**Table 1:** Classification of stakeholders among service providers of RR-TB.

**Major category**	**Classification**
Service policy implementers	Medical and nursing department of prefecture-level TB designated hospital
	Management department of prefecture-level TB designated hospital
	Management department of county-level TB designated hospital
	Medical and nursing department of county-level TB designated hospital
	Prefecture-level laboratory testing department
	Community follow-up management department
	County-level laboratory testing department
	Department of TB prevention and control of provincial CDC
	Department of TB prevention and control of provincial of county level CDC
	Department of TB prevention and control of provincial of prefecture-level CDC
Policy makers	Administrative department of health
	Department of medical insurance.
	Administrative department of civil affairs
	Administrative department of finance
Others	Charitable NGOs
	Manufacturers

### Survey Participants

Based on the list of stakeholders, we identified the research participants regarding RR-TB screening policy through a preliminary survey in Ningbo, Zhejiang Province. Afterwards, a stratified cluster sampling method was used to select stakeholders from 14 counties and seven prefectures in three provinces (Zhejiang, Hunan, and Sichuan) of eastern, central, and western China, by geographic and economic levels. In each province, two prefectures were selected according to economic level, and in each prefecture, two counties were selected according to economic level (to increase the amount of sample information, in Zhejiang Province, more prefectures and counties were selected). Representatives from these stakeholders in each selected county and prefecture were chosen as the respondents of the questionnaire survey. We trained CDC personnel in each province and prefecture to contact stakeholders and invite them to answer questionnaires.

### Data Collection, Quality Assurance, and Ethical Consent

In the returned stakeholder questionnaires, invalid questionnaires were eliminated due to missing items, logic, and other issues. In total, 245 questionnaires from stakeholders among the service providers in three provinces were collected, and questionnaire items including 8 questions on the three RR-TB screening sections were analyzed (Table 2). The respondents were investigated to understand their perceptions, attitudes, and interests on the RR-TB screening methods and models. The quantitative evaluation of their responses was rated on a 5-point Likert scale (1 = strongly disagree, 5 = strongly agree), and the survey was conducted by qualified investigators trained in each province. The questionnaire was self-administered. The quality control of each provincial survey was conducted by provincial-level investigators, and the questionnaires were returned for unified double entry. The study was approved by the ethic board of Zhejiang Provincial CDC and the questionnaire survey strictly followed the principle of informed consent.

**Table 2:** Question items involved in RR-TB screening of stakeholders among service providers.

**RR-TB screening**	**Question items**
Screening subjects selection	Whether to endorse screening only for patients with highly suspected RR-TB (rifampin-resistant high-risk)
	Whether to endorse screening for all patients with infectious (the bacteria were being discharged) TB
	Whether to endorse screening for all TB patients
Application of rapid diagnostic technologies	Whether the rapid diagnostic technologies should be actively promoted
	Whether the screening cost for new diagnostic technologies (such as rapid molecular test) is mainly reimbursed by medical insurance
	Whether the screening cost for new diagnostic technologies is mainly subsidized by financial assistance
Implementation levels of RR-TB screening	Whether RR-TB screening should be conducted at the county level
	Whether RR-TB screening should be conducted at the prefectural level

### In-depth Interview

We conducted in-depth interviews among stakeholders from provincial and prefecture-level institutions, to understand their main attitudes toward various policies or strategies for the prevention and treatment of MDR-TB. Eight individuals who represented for the three major categories of stakeholders were interviewed. The interview topics encompassed knowledge of MDR-TB, the selection of people to be screened, application of rapid diagnostic technologies and preference for MDR-TB screening levels.

### Statistical Analysis

The policy issues for the screening of RR-TB were refined and analyzed in accordance with the results of the investigation. Data were double-entered using EpiData version (Jens M. Lauritsen, Odense, Denmark), and then exported to IBM SPSS Statistics for Windows, version 18.0 (SPSS Inc., Chicago, Ill., USA) for analysis. Descriptive data were summarized and presented as frequencies and percentages. Non-parametric tests were used to analyze the differences between different attitudes toward RR-TB screening strategies, and comparisons between multiple groups of study participants were performed by Kruskal–Wallis one-way ANOVA. Tests for significance were two-tailed, and statistical significance was set at a *P* < 0.05.

## Results

### Socio-Demographic Characteristics of the Stakeholders

Among the 245 stakeholder respondents, the proportions of males (48.20%) and females (51.80%) were similar. The highest educational level of most of the respondents was college/undergraduate degree (89.00%). Of the 245 stakeholders, 58 (34.10%) had professional and technical titles (vice-high or above). About half of them (*n* = 63, 48.10%) were senior staff members at the administrative level, and most (*n* = 186, 75.90%) have worked for more than 5 years. Approximately 63 and 30% of the stakeholders were in the business and personnel sectors of the administrative departments, respectively. Among the stakeholders, service policy implementers, policy makers, and others, respectively, accounted for 64.49, 28.98, and 6.53% (Table 3).

**Table 3:** Socio-demographic characteristics of the stakeholders.

**Item**	**Classification**	**Number of people**	**Percentage (%)**
Gender	Male	118	48.20
	Female	127	51.80
Educational background	High school (technical secondary school and below)	2	0.80
	College/Undergraduate	218	89.00
	Master/Doctor	25	10.20
Professional	Senior	18	10.60
technical title	Vice-High	40	23.50
	Intermediate	64	37.60
	Junior	48	28.20
Administrative level	Clerk/staff member	24	18.30
	Senior staff member	63	48.10
	Principle staff member	40	30.50
	Division director and above	4	3.10
Working time (years)	<5	59	24.10
	5	55	22.40
	8	66	26.90
	>13	65	26.60
Roles of surveyed stakeholders	Medical staff in designated hospitals	90	37.00
	Designated hospital managers	31	12.80
	CDC business personnel	35	14.40
	Health administration personnel	20	8.20
	Financial department personnel	13	5.30
	Personnel of medical insurance department	15	6.20
	Civil affairs department staff	23	9.50
	Others	16	6.60
Stakeholder	Service policy implementers	158	64.49
categories	Policy makers	71	28.98
	Others	16	6.53

### The Attitudes Toward RR-TB Screening Strategies

#### Service Provider Stakeholder Attitudes Regarding the Selection Strategies of the Patients for the RR-TB Screening

With regards to choosing whom to provide RR-TB screening for, 66.12 and 24.49% of the stakeholders among service providers strongly and basically agreed, respectively, that they preferred to provide RR-TB screening for all patients with infectious TB (the bacteria were being discharged). Approximately 79.10% of respondents supported providing RR-TB screening to all TB patients. However, RR-TB screening for all patients with highly suspected RR-TB (drug-resistant high-risk) were strongly and basically agreed on, with >60% support. Statistically significant difference was observed between the stakeholders' attitudes toward the screening of patients (*Z* = 98.477; *P* < 0.001) (Table 4).

**Table 4:** Service provider stakeholder attitudes regarding the selection strategies of the subjects for the RR-TB screening.

**Screening subjects**	**Attitudes of stakeholders**					***Z***	***P***
	**Strongly agree**	**Basically agree**	**Neutral**	**Basically disagree**	**Strongly disagree**		
	**(%)**	**(%)**	**(%)**	**(%)**	**(%)**		
Patients with highly suspected RR-TB	68 (27.87)	64 (26.23)	63 (25.82)	37 (15.16)	12 (4.92)	98.477	<0.001
Patients with infectious (the bacteria were being discharged) TB	162 (66.12)	60 (24.49)	12 (4.90)	8 (3.27)	3 (1.22)		
All TB patients	117 (47.95)	76 (31.15)	37 (15.16)	9 (3.69)	5 (2.05)		

#### Attitudes of Different Categories of Stakeholders Among Service Providers Toward RR-TB Screening of Patients

When the attitudes of the stakeholders regarding screening for patients with highly suspected RR-TB were evaluated, the agreement level among service policy implementers, policy makers, and others was not high (about 50%). Regarding the screening of all patients with infectious TB, the service policy implementers (92.40%), policy makers (87.32%), and others (87.50%) expressed a high degree of consent. For the screening of all TB patients, the service policy implementers (82.17%), policy makers (70.43%), and others (87.50%) also expressed a relatively high degree of agreement. However, there was no significant difference between the attitudes of different stakeholders in the selection of patients for RR-TB screening (Table 5).

**Table 5:** Attitudes of different categories of stakeholders among service providers toward RR-TB screening subjects.

**Screening subjects**	**Attitudes of stakeholders**					***Z***	***P***
	**Strongly agree**	**Basically agree**	**Neutral)**	**Basically disagree**	**Strongly disagree**		
	**(%)**	**(%)**	**(%)**	**(%)**	**(%)**		
**Screening only for patients with highly suspected RR-TB (drug-resistant high-risk)**							
Service policy implementers	44 (28.03)	40 (25.48)	38 (24.20)	26 (16.56)	9 (5.73)	0.386	0.825
Policy makers	19 (26.76)	21 (29.58)	21 (29.58)	8 (11.27)	2 (2.82)		
Others	5 (31.25)	3 (18.75)	4 (25.00)	3 (18.75)	1 (6.25)		
**Screening for all patients with infectious (the bacteria were being discharged) TB**							
Service policy implementers	110 (69.62)	36 (22.78)	8 (5.06)	3 (1.90)	1 (0.63)	2.950	0.229
Policy makers	43 (60.56)	19 (26.76)	3 (4.23)	4 (5.63)	2 (2.82)		
Others	9 (56.25)	5 (31.25)	1 (6.25)	1 (6.25)	0(0.00)		
**Screening for all TB patients**							
Service policy implementers	80 (50.96)	49 (31.21)	22 (14.01)	3 (1.91)	3 (1.91)	5.183	0.075
Policy makers	28 (39.44)	22 (30.99)	13 (18.31)	6 (8.45)	2 (2.82)		
Others	9 (56.25)	5 (31.25)	2 (12.50)	0 (0.00)	0 (0.00)		

### The Attitudes Toward RR-TB Screening Methods

#### Attitudes of Stakeholders Among Service Providers Toward Strategies for the Application of RR-TB Rapid Diagnostic Technologies

Most stakeholders among service providers (87.71%) agreed that the rapid diagnostic technologies should be actively promoted, and 60.25% were strongly in favor. Statistically significant differences were found between the attitudes of stakeholders among service providers regarding whether the funding for rapid diagnostic technology screening should be reimbursed by the health insurance or directly subsidized by financial assistance (*Z* = 4.142, *P* < 0.001), with a higher percentage supporting the cost of screening for new diagnostic technologies being financially subsidized (88.98 > 79.60%) (Table 6).

**Table 6:** Attitudes of stakeholders among service providers toward strategies for the application of RR-TB rapid diagnostic technologies.

**Items**	**Attitudes of stakeholders**					***Z***	***P***
	**Strongly agree**	**Basically agree**	**Neutral**	**Basically disagree**	**Strongly disagree**		
	**(%)**	**(%)**	**(%)**	**(%)**	**(%)**		
Rapid diagnostic technologies should be actively promoted	147 (60.25)	67 (27.46)	21 (8.61)	9 (3.69)	0 (0.00)	4.142	<0.001
Screening cost is mainly reimbursed by medical insurance	133 (54.29)	62 (25.31)	32 (13.06)	16 (6.53)	2 (0.82)		
Screening cost is mainly subsidized by financial assistance	151 (61.63)	67 (27.35)	22 (8.98)	4 (1.63)	1 (0.41)		

#### Attitudes of Different Categories of Stakeholders Among Service Providers Toward the Application of RR-TB Rapid Diagnostic Technology Strategy

The analysis of the attitudes of different stakeholder categories among service providers toward the three application strategies of RR-TB rapid diagnostic technologies revealed differences (*Z*_1_= 7.538, *P* = 0.023, *Z*_2_= 12.779, *P* = 0.002, *Z*_3_= 13.865, *P* = 0.001). Pairwise comparisons showed a statistically significant difference in attitudes between the service policy implementers and policy makers (*Z* = 2.618, *P* = 0.027) regarding actively promoting rapid diagnostic technologies. The support rate of the policy makers was lower than that of the service policy implementers (77.47 <91.72%). There was a statistically significant difference in the attitudes between service policy implementers and policy makers regarding the issue of reimbursement of screening costs for new diagnostic technologies by medical insurance (*Z* = 3.574, *P* = 0.001), with lower support from policy makers than from service policy implementers (70.42 <84.18%). Regarding the issue of reimbursement of screening costs for new diagnostic technologies by financial assistance, there was a significant difference between the attitude of service policy implementers and policy makers (*Z* = 3.717, *P* = 0.001), with lower support of the policy makers than the service policy implementers (78.87 <93.04%) (Table 7).

**Table 7:** Attitudes of different categories of stakeholders among service providers toward the application of RR-TB rapid diagnostic technology strategy.

**Screening subjects**	**Attitudes of stakeholders**					***Z***	***P***
	**Strongly agree**	**Basically agree**	**Neutral**	**Basically disagree**	**Strongly disagree**		
	**(%)**	**(%)**	**(%)**	**(%)**	**(%)**		
**Rapid diagnosis technologies should be actively promoted**							
Service policy implementers	101 (64.33)	43 (27.39)	9 (5.73)	4 (2.55)	0 (0.00)	7.538	0.023
Policy makers	35 (49.30)	20 (28.17)	11 (15.49)	5 (7.04)	0 (0.00)		
Others	11 (68.75)	4 (25.00)	1 (6.25)	0 (0.00)	0 (0.00)		
**Screening cost of new diagnostic technology is mainly reimbursed by medical insurance**							
Service policy implementers	98 (62.03)	35 (22.15)	17 (10.76)	7 (4.43)	1 (0.63)	12.779	0.002
Policy makers	26 (36.62)	24 (33.80)	13 (18.31)	7 (9.86)	1 (1.41)		
Others	9 (56.25)	3 (18.75)	2 (12.50)	2 (12.50)	0 (0.00)		
**Screening cost of new diagnostic technologies is mainly subsidized by financial assistance**							
Service policy implementers	109 (68.99)	38 (24.05)	8 (5.06)	2 (1.27)	1 (0.63)	13.865	0.001
Policy makers	32 (45.07)	24 (33.80)	13 (18.31)	2 (2.82)	0 (0.00)		
Others	10 (62.50)	5 (31.25)	1 (6.25)	0 (0.00)	0 (0.00)		

### The Attitudes Toward Implementation Levels of RR-TB Screening

#### Attitudes of Stakeholders Among Service Providers Toward the Implementation Levels of RR-TB Screening

The attitudes of stakeholders among service providers toward the level at which RR-TB screening should be conducted are shown in Table 8. There was more support for RR-TB screening at the prefectural level (71.90 > 61.06%), with statistically significant differences in the respondents' attitudes toward the implementation levels of RR-TB screening (*Z* = 2.380, *P* = 0.017). Regarding RR-TB screening at the county level, by analyzing the attitudes of stakeholders in different provinces, 81.8% of the eastern region stakeholders, 79.7% of the western region stakeholders, and 11.4% of the central region stakeholders basically agreed or strongly agreed, and the difference was statistically significant (*Z* = 99.427, *P* < 0.001). Compared to eastern and western regions, the difference mainly stemmed from the central region, Hunan Province (*Z*_1_= 8.012, *P* < 0.001, *Z*_2_= 9.323, *P* < 0.001), showing a lower consent rate. Regarding RR-TB screening at the prefectural level, the differences between eastern and central regions (*Z* = 5.709, *P* < 0.001), western and central regions (*Z* = 3.420, *P* = 0.002) were significantly different.

**Table 8:** Attitudes of stakeholders among service providers toward the implementation levels of RR-TB screening.

**Implementation levels of RR-TB screening**	**Attitudes of stakeholders**					***Z***	***P***
	**Strongly agree**	**Basically agree**	**Neutral**	**Basically disagree**	**Strongly disagree**		
	**(%)**	**(%)**	**(%)**	**(%)**	**(%)**		
County level	94 (38.52)	55 (22.54)	58 (23.77)	28 (11.48)	9 (3.69)	2.380	0.017
Prefectural level	117 (48.35)	57 (23.55)	36 (14.88)	25 (10.33)	7 (2.89)		

#### Attitudes of Different Categories of Stakeholders Among Service Providers Toward Implementation Levels of RR-TB Screening

Table 9 summarizes the attitudes of different categories of stakeholders among service providers toward the level at which RR-TB screening should be implemented. At least half of the service policy implementers (61.39%), policy makers (62.86%), and others (50.00%) were in favor of RR-TB screening at the county level. Most stakeholders among service policy implementers (75.00%), policy makers (65.72%), and others (68.75%) supported the performance of RR-TB screening at the prefectural level. No significant difference in the attitude of stakeholders among service providers was noted regarding the level at which RR-TB screening should be conducted.

**Table 9:** Attitudes of different categories of stakeholders among service providers toward implementation levels of RR-TB screening.

**Screening subjects**	**Attitudes of stakeholders**					***Z***	***P***
	**Strongly agree (%)**	**Basically agree (%)**	**Neutral (%)**	**Basically disagree (%)**	**Strongly disagree (%)**		
**RR-TB screening should be conducted at the county level**							
Service policy implementers	61 (38.61)	36 (22.78)	33 (20.89)	20 (12.66)	8 (5.06)	0.260	0.878
Policy makers	27 (38.57)	17 (24.29)	18 (25.71)	8 (11.43)	0 (0.00)		
Others	6 (37.50)	2 (12.50)	7 (43.75)	0 (0.00)	1 (6.25)		
**RR-TB screening should be conducted at the prefectural level**							
Service policy implementers	80 (51.28)	37 (23.72)	22 (14.10)	14 (8.97)	3 (1.92)	2.494	0.287
Policy makers	29 (41.43)	17 (24.29)	13 (18.57)	9 (12.86)	2 (2.86)		
Others	8 (50.00)	3 (18.75)	1 (6.25)	2 (12.50)	2 (12.50)		

### Results of In-depth Interviews

Through interviews with 8 different categories of stakeholders among service providers, it was found that the stakeholder represented for policy makers had little knowledge of MDR-TB and their knowledge of its hazards was also limited. They were mostly concerned about the increase of government expenditure cost caused by the promotion of rapid diagnostic technologies, a staff of Department of Medical Insurance said, “*the current rapid diagnosis technologies of MDR-TB was not included in the medical insurance catalog. We feared that the inclusion of the catalog would cause too much pressure on the health insurance fund.”* The representatives of Service providers advocated promoting rapid diagnosis technologies and expanding the screening subjects as much as possible, A member from a prefectural CDC said, “*only by extending the rapid diagnosis technologies to more people could we find TB faster and reduce the transmission, but whether it could be successfully promoted depended on the government investment*.” As for the levels of screening, the interviewees believed that it mainly depended on the allocation of resources, “*the screening of eastern economic region should be at the county level*.”

## Discussion

In this study, the stakeholders among service provider in Zhejiang, Hunan, and Sichuan Provinces were investigated to understand their perceptions, attitudes, and interests in RR-TB screening methods and models, and to evaluate the current RR-TB screening strategy in China, to provide reference for RR-TB screening and recommendations for sustainable development.

Among the categories we studied, stakeholders among service providers were mainly divided into three parts, including policy implementers, policy makers, and others. The policy makers were at the top of the service provider stakeholder system, and they were considered as designers and planners who coordinated the policy-making progress. The policy implementers, as the practitioners, took charge of patient diagnosis, treatment, and follow-up; they also reported the drug-resistant case information. The Red Cross and other charitable organizations provided subsidies and assistance in their treatment to the RR-TB patients. Although they had no direct contact with patients, they also played a role in the screening of RR-TB. Related manufacturers should produce and provide diagnostic equipment and reagents, anti-TB drugs and other necessary products for RR-TB patients.

From our study, stakeholders among the service provider were more inclined to provide RR-TB screening services for all patients with infectious TB (the bacteria were being discharged), which was inconsistent with the current strategy of RR-TB screening for high-risk patients in most regions of the world when the study was conducted (15). The result of the analysis of the attitudes regarding RR-TB screening indicated the feasibility of screening all patients with infectious TB or all TB patients. However, increasing the number of patients screened implied a higher financial and human investment. This would require positive financial attitudes from health care workers, and sufficient financial and technical support should be provided. Therefore, the attitudes of stakeholders among service policymakers were not positive in this study.

Significant differences in the attitudes of policy implementers, policy makers, and others were shown in the application of RR-TB diagnostic technologies. The policy implementers and others were more supportive of actively promoting rapid diagnostic technologies, and that the screening costs be reimbursed by the medical insurance or subsidized by financial assistance. The difference might be focused on the cost, which is currently an important issue in the TB screening process, because the cost would enlarge the investment of resource from the corresponding stakeholders. Several studies from China had shown that the cost of TB influences the patients' health-seeking behavior, because of the high financial burden (11, 16–19). Walzl et al. (20) also identified the need to consider the cost of the evaluation and distribution of new diagnostic technologies, including impact assessment and cost-benefit studies. There was a strong association of changes in the national TB incidence and national socioeconomic indices with the general health status of the population (21). More investment would help for the RR-TB screening, however, as the main supporters of funding, updating screening technology which required more funding was also a concern for them.

Drug resistance screening is an important tool for understanding the prevalence of drug resistance in a country or region and in formulating appropriate drug-resistant TB control strategies (22). Based on the current situation of TB screening in China, RR-TB screening at county-level is believed to have a shorter path which is faster than that at higher level. However, the stakeholders in our study generally agreed that RR-TB screening should be implemented at the prefectural level. More stakeholders from eastern provinces endorse RR-TB screening at county level. To a certain extent, the selection of the implementation level of RR-TB screening is affected by regional resource allocation, which may be related to the capacity of screening personnel and equipment configuration at the prefecture and county levels. At present, China's eastern provinces were equipped with more sufficient technology, well-trained health care workers and public health infrastructures. Thus, we suggested the screening gateway could be tailored to the county-level in the eastern region, and can be promoted for the gradual expansion to the central and western regions.

This study put forward some policy measures to optimize the screening process of RR-TB in China. It suggested that the RR-TB screening should be expanded from the current scope of RR-TB high-risk population to all patients with infectious TB, and meanwhile the medical insurance and financial support were needed to reduce the economic burden of RR-TB patients. Expanding the coverage of health insurance schemes for TB outpatient services would be a key factor in reducing both the overall cost and out-of-pocket burden on patients (23). Currently, most areas in China are not equipped with rapid molecular test equipment at lower level of TB hospitals. It is necessary to promote rapid diagnosis technologies, which can significantly shorten the time to RR-TB diagnosis, reduce the waiting time of RR-TB patients, and improve their physical and mental health.

The in-depth interviews revealed the deep reasons for results of the quantitative survey. The main obstacle to the rapid diagnosis technologies promotion was resource constraints. Policy makers among stakeholders had some concerns about resource input, the possible reason was that they may not fully understood the public health threat of drug-resistant tuberculosis. Investment on screening for drug-resistant TB is not a priority option for them. Whether to screen MDR-TB at prefectural or county level mainly depended on the allocation of resources, which could be deployed at the grassroots level for implementation if conditions permitted.

This study had some limitations. First, the questionnaire survey might miss some detailed and deeper level of information. For complex questions, simple answers might not be able to capture the rich information required and thus more qualitative study would be needed for further research. Second, in the process of filling out the questionnaire, it was difficult to avoid recall bias and information loss. Due to the different survey environments, investigators' survey techniques, and questioning methods, the survey process could be affected to a certain extent. Finally, although we had selected representative stakeholders purposely by cluster sampling in different parts of China, the sample size may not be large enough when we analyze the results in different groups.

In conclusion, the study results suggest policy recommendations for RR-TB control in China. It is important to appropriately expand the screening scope of RR-TB to detect more cases. The application of rapid diagnostic technologies for RR-TB screening and the corresponding funding should be carefully considered according to the different economic conditions. Provinces with different economic status could adjust their screening implementation levels accordingly. In all, we believed considerable changes in China's National Tuberculosis Control Program are required to address these emerging challenges.

## Data Availability Statement

The original contributions presented in the study are included in the article/supplementary material, further inquiries can be directed to the corresponding author/s.

## Ethics Statement

The studies involving human participants were reviewed and approved by the Ethics Committee of the Zhejiang Provincial Center for Disease Prevention and Control. The patients/participants provided their written informed consent to participate in this study.

## Author Contributions

BC and JJ designed the study, supervised the project, and revised the manuscript. QH, HX, and XC analyzed the data, interpreted the results, produced the figures, and prepared the manuscript with support from BC and JJ. QH and XC contributed to the manuscript writing. All authors contributed to writing the manuscript and approved the final manuscript.

## Funding

This work was supported by Nation-Zhejiang Provincial Health Commission Cooperating Project (Grant no. WKJ-ZJ-2118), the Zhejiang Provincial Public Welfare Technology Research Project (Grant no. LGF19H260004), and the National Natural Science Foundation of China (Grant no. 71640019).

## Conflict of Interest

The authors declare that the research was conducted in the absence of any commercial or financial relationships that could be construed as a potential conflict of interest.

## Publisher's Note

All claims expressed in this article are solely those of the authors and do not necessarily represent those of their affiliated organizations, or those of the publisher, the editors and the reviewers. Any product that may be evaluated in this article, or claim that may be made by its manufacturer, is not guaranteed or endorsed by the publisher.
